# Caloric Restriction Extends Yeast Chronological Lifespan by Altering a Pattern of Age-Related Changes in Trehalose Concentration

**DOI:** 10.3389/fphys.2012.00256

**Published:** 2012-07-06

**Authors:** Pavlo Kyryakov, Adam Beach, Vincent R. Richard, Michelle T. Burstein, Anna Leonov, Sean Levy, Vladimir I. Titorenko

**Affiliations:** ^1^Department of Biology, Concordia UniversityMontreal, PQ, Canada

**Keywords:** yeast, cellular aging, longevity, chronological lifespan, caloric restriction, trehalose, proteostasis

## Abstract

The non-reducing disaccharide trehalose has been long considered only as a reserve carbohydrate. However, recent studies in yeast suggested that this osmolyte can protect cells and cellular proteins from oxidative damage elicited by exogenously added reactive oxygen species (ROS). Trehalose has been also shown to affect stability, folding, and aggregation of bacterial and firefly proteins heterologously expressed in heat-shocked yeast cells. Our recent investigation of how a lifespan-extending caloric restriction (CR) diet alters the metabolic history of chronologically aging yeast suggested that their longevity is programmed by the level of metabolic capacity – including trehalose biosynthesis and degradation – that yeast cells developed prior to entry into quiescence. To investigate whether trehalose homeostasis in chronologically aging yeast may play a role in longevity extension by CR, in this study we examined how single-gene-deletion mutations affecting trehalose biosynthesis and degradation impact (1) the age-related dynamics of changes in trehalose concentration; (2) yeast chronological lifespan under CR conditions; (3) the chronology of oxidative protein damage, intracellular ROS level and protein aggregation; and (4) the timeline of thermal inactivation of a protein in heat-shocked yeast cells and its subsequent reactivation in yeast returned to low temperature. Our data imply that CR extends yeast chronological lifespan in part by altering a pattern of age-related changes in trehalose concentration. We outline a model for molecular mechanisms underlying the essential role of trehalose in defining yeast longevity by modulating protein folding, misfolding, unfolding, refolding, oxidative damage, solubility, and aggregation throughout lifespan.

## Introduction

Growing evidence supports the view that the fundamental mechanisms of aging are conserved across phyla (Kenyon, 2001; Kirkwood, 2008; Fontana et al., 2010; Kenyon, 2010). The identification of single-gene mutations that extend lifespan in yeast, worms, flies, and mice revealed numerous proteins that regulate longevity (Kenyon, 2005, 2011; Fontana et al., 2010; Kaeberlein, 2010). These proteins have been implicated in a wide array of cellular processes including cell cycle, cell growth, stress response, protein folding, apoptosis, autophagy, proteasomal protein degradation, actin organization, signal transduction, nuclear DNA replication, chromatin assembly and maintenance, ribosome biogenesis and translation, lipid and carbohydrate metabolism, oxidative metabolism in mitochondria, NAD^+^ homeostasis, amino acid biosynthesis and degradation, and ammonium and amino acid uptake (Greer and Brunet, 2008; Guarente et al., 2008; Kenyon, 2010; Masoro and Austad, 2011). The spatiotemporal organization of all these numerous cellular processes and their functional states are governed by a limited number of nutrient- and energy-sensing signaling pathways that are conserved across phyla and include the insulin/insulin-like growth factor 1 (IGF-1), AMP-activated protein kinase/target of rapamycin (AMPK/TOR), and cAMP/protein kinase A (cAMP/PKA) pathways (Greer and Brunet, 2008; Narasimhan et al., 2009; Fontana et al., 2010; Kenyon, 2010).

Caloric restriction (CR), a dietary regimen in which only calorie intake is reduced but the supply of amino acids, vitamins, and other nutrients is not compromised, is known to have the most profound longevity-extending effect across phyla and to improve overall health by delaying the onset of age-related diseases (Weindruch and Walford, 1988; Masoro, 2002; Mair and Dillin, 2008; Colman et al., 2009; Anderson and Weindruch, 2010; Fontana et al., 2010). The longevity benefit associated with CR is mediated by a signaling network that integrates the insulin/IGF-1, AMPK/TOR, and cAMP/PKA longevity regulation pathways and governs a distinct group of cellular processes (Mair and Dillin, 2008; Greer and Brunet, 2009; Narasimhan et al., 2009; Fontana et al., 2010; Goldberg et al., 2010). Our recent investigation of how CR alters the metabolic history of chronologically aging yeast suggested that trehalose metabolism is one of these longevity-defining processes (Goldberg et al., 2009). A possible essential role of trehalose in regulating yeast longevity has been also suggested by other recent studies (Wang et al., 2010; Pluskal et al., 2011). Trehalose is a non-reducing disaccharide that until recently has been considered only as a reserve carbohydrate (François and Parrou, 2001). However, the demonstrated abilities of this osmolyte to protect yeast cells and cellular proteins from oxidative damage caused by exogenously added reactive oxygen species (ROS; Benaroudj et al., 2001) or inflicted in the process of industrial alcoholic fermentation (Trevisol et al., 2011) and to impact stability, folding, and aggregation of bacterial and firefly proteins heterologously expressed in heat-shocked yeast (Singer and Lindquist, 1998a,b) suggested that trehalose may exhibit similar effects on endogenous proteins in cells of yeast and other organisms (Singer and Lindquist, 1998a,b; Elbein et al., 2003; Jain and Roy, 2009, 2010). It is conceivable therefore that trehalose may be involved in modulating cellular protein homeostasis (proteostasis). By maintaining proper synthesis, post-translational modifications, folding, trafficking, degradation, and turnover of proteins within a cell, an evolutionarily conserved proteostasis network governs various cellular activities, influences diverse age-related pathologies, and defines organismal healthspan and longevity (Tavernarakis, 2010; Morimoto et al., 2012).

To evaluate a potential role of trehalose in lifespan extension by CR, in this study we monitored how single-gene-deletion mutations that alter trehalose concentrations in pre-quiescent and quiescent yeast cells affect longevity of chronologically aging yeast under CR conditions. We also elucidated how these mutations influence the chronology of oxidative protein carbonylation, intracellular ROS, protein aggregation, thermal inactivation of a protein in heat-shocked yeast cells and a subsequent reactivation of this protein in yeast shifted to low temperature. Our findings provide evidence that the longevity-extending effect of a CR diet in chronologically aging yeast is due in part to a specific pattern of age-related changes in trehalose concentration elicited by CR. Based on these findings, we propose a model for molecular mechanisms by which trehalose modulates cellular proteostasis throughout lifespan, thereby defining yeast longevity.

## Materials and Methods

### Yeast strains and growth conditions

The wild-type (WT) strain BY4742 (*MAT*α *his3Δ1 leu2Δ0 lys2Δ0 ura3Δ0*) and single-gene-deletion mutant strains in the BY4742 genetic background (all from Open Biosystems) were grown in YP medium (1% yeast extract, 2% peptone) containing 0.2% glucose as carbon source. Cells were cultured at 30°C with rotational shaking at 200 rpm in Erlenmeyer flasks at a “flask volume/medium volume” ratio of 5:1.

### Chronological lifespan assay

A sample of cells was taken from a culture at a certain time-point. A fraction of the sample was diluted in order to determine the total number of cells using a hemacytometer. Another fraction of the cell sample was diluted and serial dilutions of cells were plated in duplicate onto YP plates containing 2% glucose as carbon source. After 2 day of incubation at 30°C, the number of colony forming units (CFU) per plate was counted. The number of CFU was defined as the number of viable cells in a sample. For each culture, the percentage of viable cells was calculated as follows: (number of viable cells per ml/total number of cells per ml) × 100. The percentage of viable cells in mid-logarithmic phase was set at 100%. The lifespan curves were validated using a LIVE/DEAD yeast viability kit (Invitrogen) following the manufacturer’s instructions.

### Trehalose concentration measurement

Preparation of alkali cellular extract and a microanalytic biochemical assay for measuring trehalose concentration were performed as previously described (Lin et al., 2001). To prepare an alkali cellular extract, 2 × 10^9^ cells were harvested by centrifugation for 1 min at 21,000 × *g* at 4°C. The cells were washed three times in ice-cold PBS (20 mM KH_2_PO_4_/KOH, pH 7.5, and 150 mM NaCl). The cell pellet was quickly resuspended in 200 μl of ice-cold SHE solution (50 mM NaOH, and 1 mM EDTA), and 800 μl of ice-cold SHE solution were added to the cell suspension. The resulting alkali extract was incubated at 60°C for 30 min to destroy endogenous enzyme activities and pyridine nucleotides. The extract was neutralized by adding 500 μl of THA solution (100 mM Tris/HCl, pH 8.1, and 50 mM HCl), divided into 150-μl aliquots, quickly frozen in liquid nitrogen, and stored at – 80°C prior to use. To measure trehalose concentration, 50 μl of alkali extract (recovered from the total of 6.5 × 10^7^ cells) were added to 150 μl of trehalose reagent [25 mM KH_2_PO_4_/KOH, pH 7.5, and 0.02% BSA; with or without 15 mU trehalase (Sigma)]. The mixture was incubated for 60 min at 37°C. Eight hundred microliters of glucose reagent [100 mM Tris/HCl, pH 8.1, 2 mM MgCl_2_, 1 mM DTT, 1 mM ATP, 0.2 mM NADP^+^, and mixture of hexokinase (7 U) and glucose-6-phosphate dehydrogenase (8 U; Sigma)] was added and the mixture incubated for 30 min at 25°C. The NADPH generated from NADP^+^ was measured fluorimetrically (excitation at 365 nm, emission monitored at 460 nm).

### Hexokinase activity measurement

Preparation of cellular lysate and a microanalytic biochemical assay for measuring hexokinase enzymatic activity were performed as previously described (Lin et al., 2001). To prepare a cellular lysate, 2 × 10^7^ cells were harvested by centrifugation for 1 min at 21,000 × *g* at 4°C. The cells were washed three times in ice-cold PBS (20 mM KH_2_PO_4_/KOH, pH 7.5, and 150 mM NaCl). The cell pellet was quickly resuspended in 800 μl of EB buffer (20 mM KH_2_PO_4_/KOH, pH 7.5, 0.02% BSA, 0.5 mM EDTA, 5 mM β-mercaptoethanol, 25% glycerol, and 0.5% Triton X-100) and incubated for 5 min at 25°C. The resulting lysate was divided into 40-μl aliquots and stored at – 80°C prior to use. To measure hexokinase activity, 4 μl of cellular lysate (recovered from the total of 1 × 10^5^ cells) were added to 996 μl of hexokinase reagent [100 mM Tris/HCl, pH 8.1, 0.05% BSA, 7 mM MgCl_2_, 5 mM ATP, 5 mM glucose, 0.5 mM DTT, 100 μM NADP^+^, 0.5% Triton X-100, and 2 U glucose-6-phosphate dehydrogenase (Sigma)]. The mixture was incubated for 1 h at 25°C. The NADPH generated from NADP^+^ was measured fluorimetrically (excitation at 365 nm, emission monitored at 460 nm). To monitor the extent of thermal inactivation of hexokinase in heat-shocked yeast cells and the efficacy of its reactivation during subsequent incubation of these cells at low temperature, yeast were grown at 29°C, and recovered upon entry into a quiescent state at day 7 or following such an entry at day 13. These cells were treated with cycloheximide for 5 min at 29°C, heat-shocked for 60 min at 43°C, then shifted to 29°C, and incubated for 60 min. Hexokinase enzymatic activity was measured every 15 min of heat shock treatment and every 15 min of the following incubation at 29°C.

### Immunodetection of carbonyl groups in oxidatively damaged cellular proteins

Total cell lysates were made by vortexing the cells in ice-cold TCL buffer (25 mM MOPS/KOH, pH 7.2, 150 mM NaCl, 50 mM DTT, and 1% CHAPS) with glass beads three times for 1 min. Lysates were then centrifuged for 5 min at 21,000 × *g* at 4°C, and the supernatants of total cell lysates were collected. The carbonyl groups of proteins recovered in total cell lysates were derivatized to 2,4-dinitrophenylhydrazones using the OxyBlot™ Protein Oxidation Detection Kit (Chemicon), according to the manufacturer’s instructions. Briefly, total cellular proteins were denatured by adding 12% SDS to an equal volume of the total cell lysate containing 10 μg of protein. Denatured proteins were incubated with 2,4-dinitrophenylhydrazine for 15 min at room temperature. Proteins were separated by 12.5% SDS-PAGE. Immunoblotting using a Trans-Blot SD semi-dry electrophoretic transfer system (Bio-Rad) was performed as described (Titorenko et al., 1998). The derivatized carbonyl groups were detected with a 2,4-dinitrophenyl-specific antibody (Chemicon) and the Amersham ECL Western Blotting System (GE Healthcare).

### ROS measurement

Reactive oxygen species were measured in live yeast by fluorescence microscopy of Dihydrorhodamine 123 (DHR) staining according to established procedures (Madeo et al., 1997; Goldberg et al., 2009). Briefly, 5 × 10^6^ cells were harvested by centrifugation for 1 min at 21,000 × *g* at room temperature and then resuspended in 100 μl of PBS. DHR (Sigma) was added to a final concentration of 10 μM. Following incubation in the dark for 60 min at room temperature, the cells were washed in PBS, and then analyzed by fluorescence microscopy. Images were collected with a Zeiss Axioplan fluorescence microscope (Zeiss) mounted with a SPOT Insight 2 megapixel color mosaic digital camera (Spot Diagnostic Instruments). Fluorescence of individual DHR-positive cells in arbitrary units was determined by using the UTHSCSA Image Tool software (Version 3.0). In each of three to six independent experiments, the value of median fluorescence was calculated by analyzing at least 800–1000 cells that were collected at each time-point. The median fluorescence values were plotted as a function of the number of days cells were cultured.

### Recovery of insoluble aggregates of denatured proteins

Insoluble aggregates of denatured proteins were recovered according to established procedures (Parsell et al., 1994; Boukh-Viner et al., 2005), with the following modifications. Total cell lysates were made by vortexing the cells in ice-cold MBS buffer (25 mM MOPS/KOH, pH 7.2, and 150 mM NaCl) with glass beads four times for 1 min. Unbroken cells and cell debris were removed by centrifugation for 3 min at 1,000 × *g* at 4°C. The supernatants of total cell lysates were collected and normalized by dilution to a final concentration of 1 mg/ml. Equal aliquots of the total cell lysates were supplemented with 3-[(3-Cholamidopropyl)dimethylammonio]-1-propanesulfonate (CHAPS; Sigma) to a final concentration of 10 mM. CHAPS is a zwitterionic, non-denaturing, and electrically neutral detergent; although it protects a native state of soluble proteins and efficiently solubilizes intrinsic membrane proteins (including proteins associated with lipid raft membrane domains), it is unable to solubilize aggregates of denatured proteins (Chow and Zukin, 1983; Evans et al., 1986; Boukh-Viner et al., 2005; Tao et al., 2010). After incubation on ice for 30 min, samples were subjected to centrifugation at 100,000 × *g* for 30 min at 4°C. The pellet fractions of insoluble aggregates of denatured proteins were analyzed by 12.5% SDS-PAGE, followed by silver staining.

### Statistical analysis

Statistical analysis was performed using Microsoft Excel’s (2010) Analysis ToolPack-VBA. All data are presented as mean ± SEM. The *p* values were calculated using an unpaired two-tailed *t* test.

## Results

### Lifespan extension by CR requires a specific pattern of age-related changes in trehalose concentration

To evaluate the effect of trehalose on lifespan extension by CR, we incubated WT strain and several mutant strains, each carrying a single-gene-deletion mutation affecting trehalose biosynthesis or degradation (François and Parrou, 2001), in YP medium initially containing 0.2% glucose. We monitored the chronological lifespans of all these strains and assessed the dynamics of changes in trehalose concentration during their aging under CR conditions.

**Figure 1:**
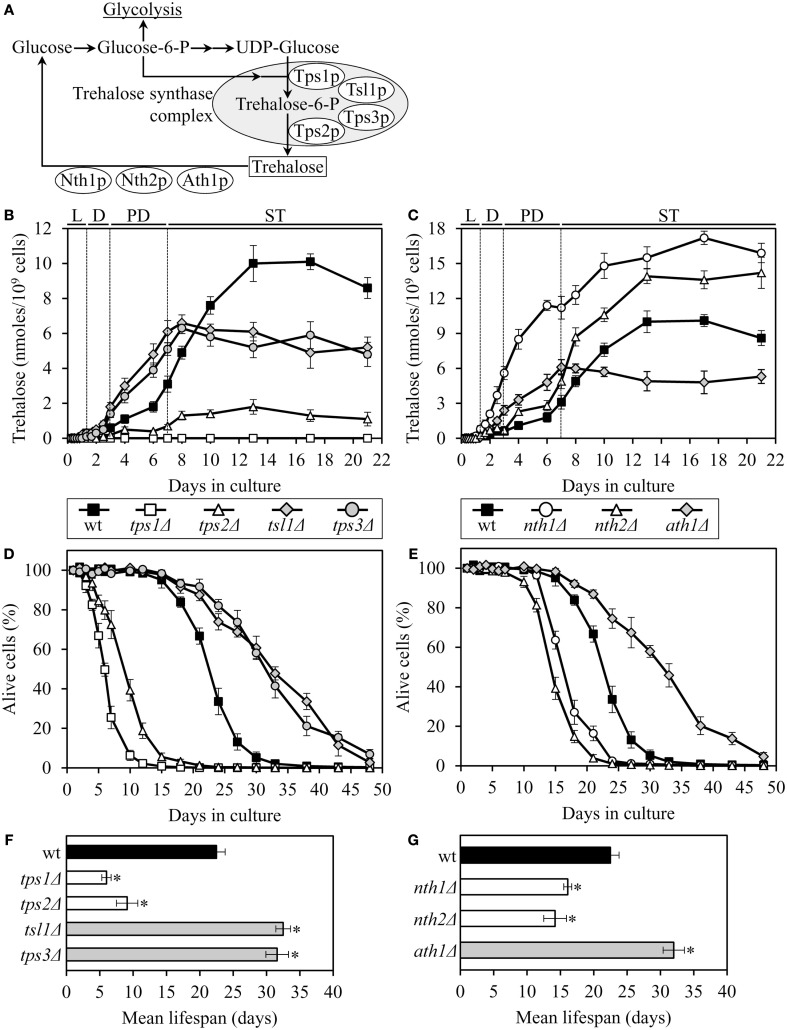
**The chronological lifespan of yeast grown under CR conditions can be extended by mutations that simultaneously increase trehalose concentration prior to quiescence and reduce trehalose concentration following entry into a quiescent state**. **(A)** Outline of metabolic pathways of trehalose biosynthesis and degradation. **(B,C)** The dynamics of age-dependent changes in the intracellular levels of trehalose during chronological aging of wild-type (wt) and mutant strains. **(D–G)** Survival **(D,E)** and the mean lifespans **(F,G)** of chronologically aging wt and mutant strains. Each mutant carried a single-gene-deletion mutation that affects trehalose biosynthesis or degradation. Cells were cultured in YP medium initially containing 0.2% glucose. Data are presented as mean ± SEM (*n* = 5–6); *p < 0.01 (relative to the mean lifespan of wt strain). Abbreviations: D, diauxic growth phase; L, logarithmic growth phase; PD, post-diauxic growth phase; ST, stationary growth phase.

The *tps1*Δ and *tps2*Δ mutations, which eliminate two different catalytic subunits of the trehalose synthase complex (Figure 1A), decreased intracellular trehalose concentration and shortened lifespan (Figures 1B,D). Yeast whose trehalose level was increased before they have entered the non-proliferative stationary (ST) growth phase and remained elevated during ST phase – as it was observed in mutant cells lacking the Nth1p isozyme of neutral trehalase – were short-lived (Figures 1C,E). Moreover, even if trehalose concentration exceeded the level seen in WT only after yeast have entered ST phase – as it occurred in mutant cells lacking the Nth2p isozyme of neutral trehalase – cells were short-lived (Figures 1C,E). Importantly, some genetic manipulations altering trehalose concentration extended lifespan. Specifically, in long-lived mutants lacking the Tsl1p or Tps3p regulatory subunit of the trehalose synthase complex, trehalose concentration exceeded that in WT until the end of post-diauxic (PD) growth phase, but then in ST phase reached a plateau at the level that was 50–70% of that in WT (Figures 1B,D). Similar dynamics of age-related changes in trehalose concentration was observed in the long-lived mutant *ath1*Δ lacking acid trehalase (Figures 1C,E).

Altogether, these findings imply that the extended chronological lifespan of CR yeast (as compared to that of non-CR yeast) can be further prolonged by genetic manipulations that simultaneously (1) increase trehalose concentration by 70–160% during PD phase, prior to entry into a quiescent state; and (2) reduce trehalose concentration by 60–80% during ST phase, following entry into quiescence. Thus, lifespan extension by a low calorie diet requires a specific pattern of age-related changes in the intracellular level of trehalose.

### Mutations that increase trehalose concentration prior to entry into quiescence reduce oxidative damage to cellular proteins throughout lifespan, irrespective of their effects on longevity

Trehalose accumulation in exponentially grown yeast cells exposed to elevated temperature or to a proteasome inhibitor has been shown to increase their ability to survive a subsequent treatment with exogenous ROS and to protect cellular proteins from oxidative carbonylation caused by such a treatment (Benaroudj et al., 2001). According to the mitochondrial free radical theory of aging, the gradual accumulation of macromolecular damage caused by mitochondrially produced ROS throughout lifespan accelerates cellular dysfunction and later in life leads to a functional decline and increased mortality (Harman, 1956, 1972). Although a body of evidence does not validate the core statement of this theory on a casual role of ROS generation in aging, the importance of ROS in mediating a stress response to age-related cellular damage is supported by numerous findings (Gems and Doonan, 2009; Pérez et al., 2009; Lapointe and Hekimi, 2010; Ristow and Zarse, 2010; Sanz et al., 2010; Hekimi et al., 2011). To evaluate a potential role of trehalose in linking a ROS-dependent oxidative macromolecular damage to lifespan extension by CR, we assessed how the *tsl1*Δ and *nth1*Δ mutations influence the dynamics of age-related changes in protein carbonylation and ROS in yeast grown under CR conditions.

**Figure 2:**
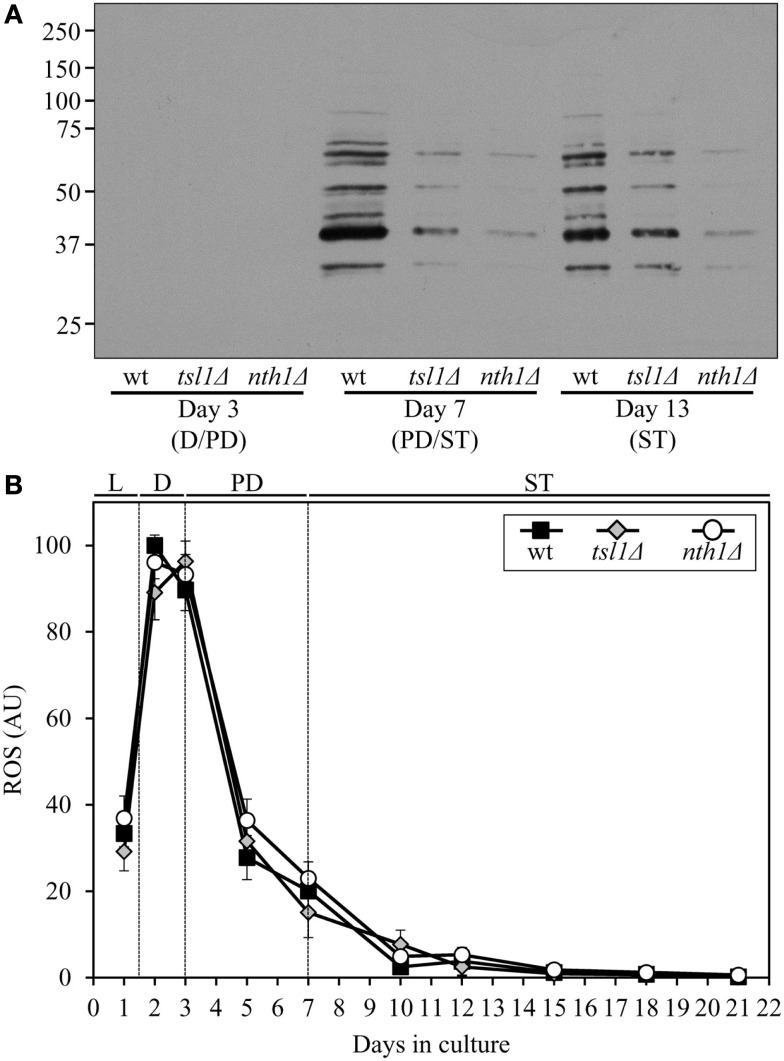
**Although mutations that in yeast grown under CR conditions increase trehalose concentration prior to entry into quiescence do not alter ROS levels, they reduce oxidative damage to cellular proteins throughout lifespan**. **(A)** Immunodetection of carbonyl groups in oxidatively damaged cellular proteins in chronologically aging wt and mutant strains. **(B)** The dynamics of age-related changes in intracellular ROS levels during chronological aging of wt and mutant strains. wt, *tsl1*Δ and *nth1*Δ cells were cultured in YP medium initially containing 0.2% glucose. Data are presented as mean ± SEM (*n* = 3–4). Abbreviations: D, diauxic growth phase; PD, post-diauxic growth phase; ST, stationary growth phase.

Both the *tsl1*Δ and *nth1*Δ mutations elevated trehalose concentration (Figures 1B,C) and reduced oxidative carbonylation of cellular proteins (Figure 2A) during PD phase, prior to entry into a quiescent state. None of these mutations altered ROS levels in pre-quiescent cells (Figure 2B). Thus, it is unlikely that the observed reduction of oxidative damage to cellular proteins in pre-quiescent *tsl1*Δ and *nth1*Δ cells was due to the previously proposed by Benaroudj et al. (2001) ability of trehalose, a non-reducing disaccharide, to quench ROS. It is conceivable therefore that prior to quiescence trehalose protects cellular proteins from oxidative carbonylation (Figure 2A) by interacting with their carbonylation-prone misfolded and unfolded species. These aberrantly folded protein species are known to be much more sensitive to oxidative carbonylation than their properly folded counterparts (Nyström, 2005; Hipkiss, 2006).

The extent of protein carbonylation reached prior to entry into a quiescent state was not significantly altered in *tsl1*Δ and *nth1*Δ cells following entry into quiescence (Figure 2A), likely due to greatly diminished ROS levels observed in quiescent *tsl1*Δ and *nth1*Δ cells (Figure 2B). Trehalose concentration in quiescent *tsl1*Δ cells was substantially lower than that seen in quiescent WT cells (Figure 1B). In contract, the concentration of trehalose in quiescent *nth1*Δ cells exceeded the level detected in quiescent WT cells (Figure 1C). We therefore concluded that genetic manipulations that increase trehalose concentration prior to entry into a quiescent state reduce oxidative damage to cellular proteins throughout lifespan, regardless of their effects on the intracellular concentration of this non-reducing disaccharide following entry into quiescence.

Although both the *tsl1*Δ and *nth1*Δ mutations reduced oxidative carbonylation of cellular proteins throughout lifespan (Figure 2A), their effects on longevity differed. The *tsl1*Δ mutation extended yeast lifespan, whereas the *nth1*Δ mutations shortened it (Figures 1D–G). Hence, it is unlikely that the observed ability of these genetic manipulations to protect cellular proteins from oxidative damage plays a role in defining yeast longevity under CR conditions.

### A pattern of age-related changes in trehalose concentration defines the dynamics of protein aggregation throughout lifespan

Trehalose has been shown to (1) stabilize bacterial and firefly luciferases in their native (folded) states in heat-shocked yeast cells; (2) prevent aggregation and maintain non-native (misfolded or partially folded) states of these two luciferases, as well as of firefly rhodanese, following their guanidinium-induced denaturation *in vitro* and in yeast cells briefly exposed to elevated temperature; and (3) inhibit the refolding and reactivation of these pre-denatured bacterial and firefly proteins *in vitro* and in yeast cells by interfering with chaperone-assisted folding of their non-native (misfolded or partially folded) species (Singer and Lindquist, 1998a). It has been predicted that trehalose may exhibit similar effects on the stability, folding, and aggregation of endogenous proteins in cells of yeast and other organisms (Singer and Lindquist, 1998a,b; Elbein et al., 2003; Jain and Roy, 2009, 2010; Mir et al., 2009). Furthermore, our investigation of how a CR diet affects the metabolic history of chronologically aging yeast suggested that the elevated level of trehalose observed prior to entry into quiescence in slowly aging CR yeast (as compared to that seen in rapidly aging non-CR yeast) protects from aggregation proteins that have been completely or partially unfolded and/or oxidatively carbonylated due to their exposure to intracellular ROS (Goldberg et al., 2009). We hypothesized that (1) such protective effect of high trehalose concentrations could contribute to the enhanced survival of CR yeast (as compared to survival of non-CR yeast) following their entry into quiescence; and (2) a dietary or genetic intervention providing yeast with the ability to maintain trehalose concentration at a certain “optimal” level prior and following entry into a quiescent state would extend their longevity (Goldberg et al., 2009). We predicted that at such an “optimal” level trehalose concentration is (1) sufficiently high prior to entry into quiescence to allow this osmolyte to prevent aggregation of proteins that have been completely or partially unfolded and/or oxidatively carbonylated; and (2) sufficiently low following entry into quiescence to reduce the efficiency with which trehalose inhibits the refolding and reactivation of partially unfolded and/or oxidatively carbonylated proteins (Goldberg et al., 2009).

**Figure 3:**
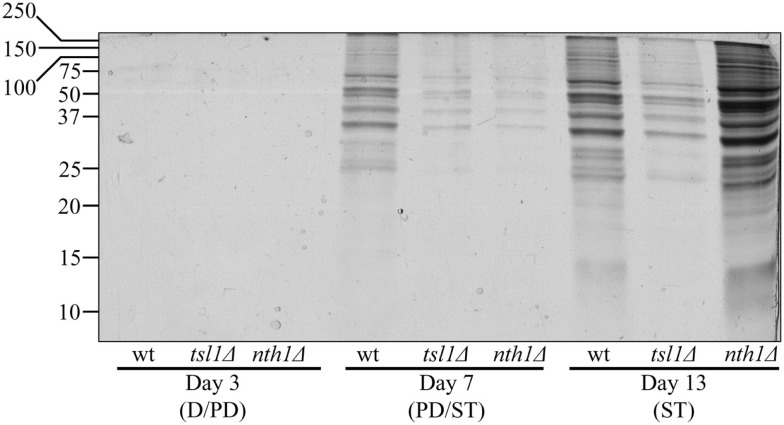
**In yeast grown under CR conditions, a pattern of age-related changes in trehalose concentration defines the dynamics of protein aggregation throughout lifespan**. Total cell lysates were made by vortexing the cells in ice-cold buffer with glass beads. Unbroken cells and cell debris were removed by centrifugation for 3 min at 1,000 × *g* at 4°C. The supernatants of total cell lysates were collected and normalized by dilution to a final concentration of 1 mg/ml. Equal aliquots of the total cell lysates were supplemented with CHAPS, a zwitterionic, non-denaturing, and electrically neutral detergent that protects a native state of soluble proteins and efficiently solubilizes membrane proteins, but is unable to solubilize aggregates of denatured proteins. After incubation on ice for 30 min, samples were subjected to centrifugation at 100,000 × *g* for 30 min at 4°C. The pellet fractions of insoluble aggregates of denatured proteins were analyzed by 12.5% SDS-PAGE, followed by silver staining. wt, *tsl1*Δ and *nth1*Δ cells were cultured in YP medium initially containing 0.2% glucose. Abbreviations: D, diauxic growth phase; PD, post-diauxic growth phase; ST, stationary growth phase.

To test the validity of our hypothesis, we assessed how the *tsl1*Δ and *nth1*Δ mutations influence the dynamics of age-related changes in the extent of protein aggregation in yeast limited in calories. We found that both these mutations, which we demonstrated to elevate trehalose concentration (Figures 1B,C) and to decrease oxidative protein carbonylation (Figure 2A) during PD phase, significantly reduce the extent of aggregation of cellular proteins during this growth phase preceding entry into a quiescent state (Figure 3). Following entry into quiescence, the extent of protein aggregation in *tsl1*Δ cells was substantially lower than that seen in quiescent WT cells and especially in quiescent *nth1*Δ cells (Figure 3). As we mentioned above, trehalose concentration in quiescent *tsl1*Δ cells was significantly reduced as compared to that in WT (Figure 1B) and especially in *nth1*Δ (Figure 1C) cells reached reproductive maturation. Furthermore, both the concentration of trehalose (Figures 1B,C) and the extent of protein aggregation (Figure 3) in quiescent *nth1*Δ cells were significantly higher than that observed in WT cells and especially in *tsl1*Δ cells entered a quiescent state.

In sum, these findings validate our hypothesis in which a genetic intervention will extend longevity of calorically restricted yeast if it (1) elevates trehalose concentration prior to entry into quiescence to allow this osmolytic disaccharide to prevent aggregation of completely or partially unfolded and/or oxidatively carbonylated cellular proteins; and (2) reduces the concentration of trehalose following entry into quiescence to limit its inhibitory effect on the refolding and reactivation of partially unfolded and/or oxidatively carbonylated proteins.

### Trehalose concentration in yeast cells defines the sensitivity of an endogenous enzyme to thermal inactivation and the extent of its subsequent reactivation at low temperature

**Figure 4:**
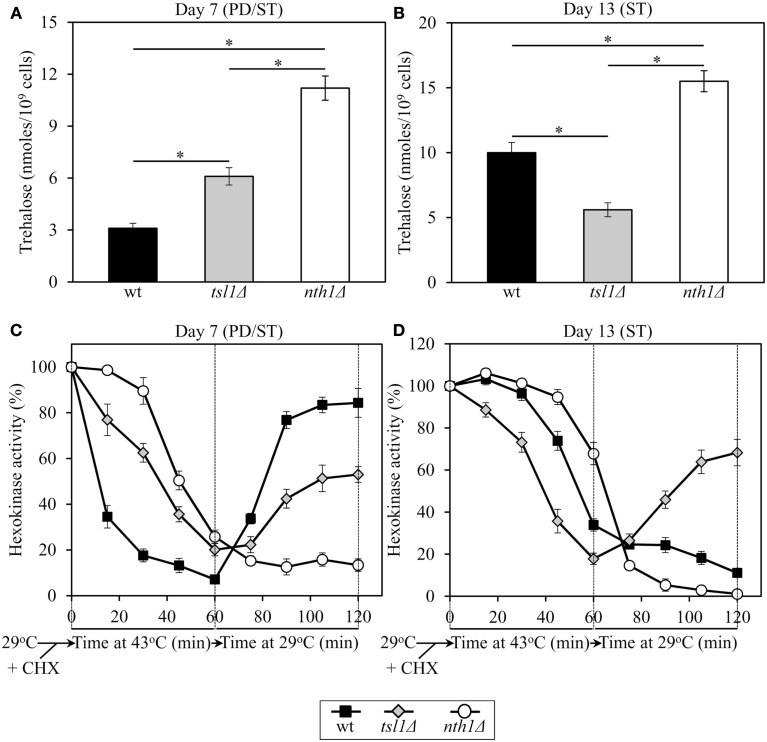
**In yeast grown under CR conditions, trehalose concentration defines the sensitivity of hexokinase, an endogenous enzyme, to thermal inactivation, and the extent of its subsequent reactivation at low temperature**. Yeast cells grown at 29°C were recovered upon entry into a quiescent state at day 7 or following such an entry at day 13. The cells were treated with cycloheximide for 5 min at 29°C to inhibit protein synthesis, heat-shocked for 60 min at 43°C, then shifted to 29°C, and incubated for 60 min. **(A,B)** The intracellular levels of trehalose prior to cell treatment with cycloheximide. **(C,D)** Changes in hexokinase enzymatic activity following cell exposure to cycloheximide, during heat shock treatment for 60 min at 43°C, and subsequent incubation for 60 min at 29°C. wt, *tsl1*Δ and *nth1*Δ cells were cultured in YP medium initially containing 0.2% glucose. Data are presented as mean ± SEM (*n* = 3–5). Abbreviations: CHX, cycloheximide; PD, post-diauxic growth phase; ST, stationary growth phase.

To use a complementary experimental approach for validating our hypothesis on a longevity-defining role of trehalose concentration in maintaining biological activities of proteins in chronologically aging yeast under CR conditions, we assessed how the *tsl1*Δ and *nth1*Δ mutations influence (1) the extent of thermal inactivation of hexokinase, an endogenous enzyme protein, in heat-shocked yeast cells; and (2) the efficacy of its reactivation during subsequent incubation of these cells at low temperature. In these experiments, yeast grown at 29°C and recovered upon entry into a quiescent state or following such an entry were treated with cycloheximide for 5 min at 29°C, heat-shocked for 60 min at 43°C, then shifted to 29°C, and incubated for 60 min (Figure 4).

In *tsl1*Δ and *nth1*Δ cells recovered at day 7, upon entry into a quiescent state, the activity of hexokinase synthesized prior to a cycloheximide-induced inhibition of protein synthesis at 29°C was less susceptible to thermal inactivation at 43°C than in identically treated and aged WT cells (Figure 4C). Under these conditions, trehalose concentrations in both *tsl1*Δ and *nth1*Δ cells exceeded that in WT cells of the same age (Figure 4A). If cells were recovered at day 13, following entry into a quiescent state, hexokinase activity in *tsl1*Δ cells having a lower trehalose concentration than WT cells (Figure 4B) was more susceptible to the thermal inactivation at 43°C then in WT cells (Figure 4D). In contrast, in *nth1*Δ cells recovered at day 13 and having a higher trehalose concentration then WT cells of the same age (Figure 4B) hexokinase activity was less susceptible to such thermal inactivation then in WT cells (Figure 4D). These findings imply that in calorically restricted pre-quiescent yeast trehalose preserves biological activities of partially inactivated cellular proteins, perhaps by stabilizing their native (folded) state, preventing their unfolding, and/or inhibiting their subsequent aggregation.

In *tsl1*Δ and *nth1*Δ cells recovered at day 7, upon entry into quiescence, the reactivation of thermally inactivated hexokinase during the subsequent incubation at low temperature occurred less efficient then in WT cells of the same age (Figure 4C). Noteworthy, the efficacy of such hexokinase reactivation was inversely proportional to trehalose concentration in yeast cells that reached a transition to a quiescent state (Figures 4A,C). If cells were recovered at day 13, following entry into quiescence, the reactivation of thermally inactivated hexokinase during the subsequent incubation at low temperature occurred only in *tsl1*Δ cells having lower trehalose concentration as compared to WT and especially to *nth11*Δ cells of the same age (Figures 4B,D). In *nth1*Δ cells recovered at day 13 and having a higher trehalose concentration then WT cells of the same age (Figure 4B), thermally inactivated hexokinase was further inactivated during the subsequent incubation at low temperature with the efficiency exceeding that in WT cells (Figure 4D). These findings imply that in calorically restricted quiescent yeast trehalose inhibits the reactivation of inactivated cellular proteins, perhaps by interfering with chaperone-assisted folding of their non-native (misfolded or partially folded) species.

## Discussion

**Figure 5:**
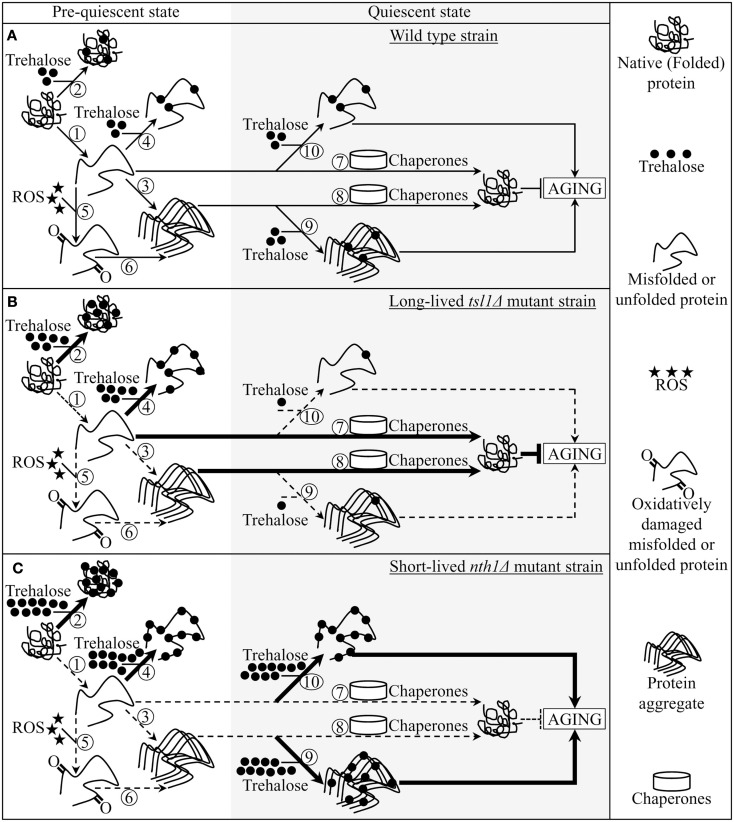
**A model for molecular mechanisms underlying the essential role of trehalose in defining yeast longevity by modulating cellular proteostasis throughout lifespan**. The outlined model adequately explains how the *tsl1*Δ and *nth1*Δ mutations altering a pattern of age-related changes in trehalose concentration influence a longevity-defining balance between protein folding, misfolding, unfolding, refolding, oxidative damage, solubility, and aggregation. **(A–C)** The effects of trehalose on essential processes governing proteostasis in wild-type **(A)**, *tsl1Δ* **(B)** and *nth1Δ* **(C)** cells prior to and following entry into a quiescent state are outlined. See text for details. The thickness of arrows and T bars correlates with the rates of the processes taking place in chronologically aging yeast prior to entry into a quiescent state and following such an entry under CR conditions. T bars denote inhibition of the process. Abbreviation: ROS, reactive oxygen species.

To investigate whether trehalose homeostasis in yeast cells may play a role in longevity extension by CR, we assessed how single-gene-deletion mutations that in chronologically aging yeast alter trehalose concentrations prior to quiescence and following entry into a quiescent state impact lifespan. We also examined the effects of these mutations on the chronology of oxidative protein carbonylation, intracellular ROS, protein aggregation, thermal inactivation of a protein in heat-shocked yeast cells and a subsequent reactivation of this protein in yeast shifted to low temperature. Our findings provide evidence that CR extends yeast chronological lifespan in part by altering a pattern of age-related changes in trehalose concentration. Based on our data, we propose a model for molecular mechanisms underlying the essential role of trehalose in defining yeast longevity by modulating cellular proteostasis throughout lifespan (Figure 5). This outlined below model adequately explains how genetic interventions altering a pattern of age-related changes in trehalose concentration influence a longevity-defining balance between protein folding, misfolding, unfolding, refolding, oxidative damage, solubility, and aggregation.

Pre-quiescent WT cells proliferating under CR conditions cope with a flow of misfolded, partially folded, and unfolded protein species in the non-native folding state (Figure 5A, process 1). The continuous formation of these protein species within a proliferating cell is due to a number of factors, including (1) macromolecular crowding, which is caused by the very high intracellular protein concentration and leads to inappropriate intermolecular contacts; (2) stochastic fluctuations in protein structure; (3) transcriptional errors; (4) inherited genetic polymorphisms, including gene copy number variations; (5) intrinsic errors in gene expression that may create an excess of unassembled subunits of oligomeric protein complexes; (6) errors in protein translation – such as missense incorporation of amino acids, frame-shifting, stop-codon readthrough, and premature termination; (7) defects in post-translational protein modifications and turnover; and (8) inefficient translocation of secretory and mitochondrial precursor proteins across membranes of their target organelles (Chen et al., 2011; Gidalevitz et al., 2011; Lindquist and Kelly, 2011). In pre-quiescent WT cells, trehalose stabilizes the native state of proteins and thereby reduces the formation of their aberrantly folded species (Figure 5A, process 2). The promoted by trehalose shift of a balance between native and non-native protein folding states toward properly folded protein species is amplified by the *tsl1*Δ and *nth1*Δ mutations, both of which significantly elevate trehalose concentration prior to entry into a quiescent state (Figures 5B,C, process 2). Our finding that the enzymatic activity of an endogenous hexokinase synthesized prior to a cycloheximide-induced inhibition of protein synthesis at 29°C in pre-quiescent *tsl1*Δ and *nth1*Δ cells is significantly less susceptible to thermal inactivation at 43°C than in identically treated and chronologically aged WT cells (Figure 4C) supports the role of trehalose in stabilizing the native state of cellular proteins. Moreover, a previously demonstrated ability of trehalose to stabilize bacterial and firefly luciferases in their native states in heat-shocked yeast cells (Singer and Lindquist, 1998a) provides additional support for the validity of our conclusion on the essential role of this osmolyte in shifting a balance between native and non-native protein folding states toward native folding structures. It is conceivable that trehalose may stabilize the native state of proteins in pre-quiescent yeast cells via any of the three recently proposed mechanisms (Jain and Roy, 2009, 2010).

In pre-quiescent WT cells, the aberrantly folded protein species that have not been refolded into functional three-dimensional native conformations or degraded within an elaborate network of molecular chaperones and protein degradation factors (Chen et al., 2011; Gidalevitz et al., 2011; Lindquist and Kelly, 2011) form insoluble aggregates (Figure 5A, process 3). In these cells, trehalose reduces the formation of such protein aggregates, perhaps by shielding the contiguous exposed hydrophobic side chains of amino acids that are abundant in misfolded, partially folded, and unfolded protein species and promote their aggregation (Figure 5A, process 4). Our finding that the *tsl1*Δ and *nth1*Δ mutations, both of which elevate trehalose concentration prior to entry into quiescence (Figures 1B,C), significantly reduce the extent of protein aggregation in pre-quiescent cells (Figure 3) supports the essential role of trehalose in preventing the formation of insoluble protein aggregates in these proliferation-competent cells (Figures 5B,C, process 4). Moreover, a previously demonstrated ability of trehalose to prevent aggregation and maintain non-native states of bacterial and firefly luciferases, as well as of firefly rhodanese, following their guanidinium-induced denaturation *in vitro* and in heat-shocked yeast cells (Singer and Lindquist, 1998a) further validates our conclusion that trehalose inhibits aggregation of the aberrantly folded protein species accumulating in pre-quiescent yeast.

The misfolded, partially folded, and unfolded protein species present in pre-quiescent WT cells are known to be more sensitive to ROS-driven oxidative carbonylation than their properly folded counterparts (Nyström, 2005; Hipkiss, 2006). These cells accumulate substantial levels of ROS (Figure 2B), which oxidatively damage a pool of the aberrantly folded and unfolded proteins (Figure 2A) prior to entry into a quiescent state (Figure 5A, process 5). Prior to quiescence, trehalose protects cellular proteins from oxidative carbonylation by interacting with their carbonylation-prone misfolded and unfolded species (Figure 5A, process 4) but not by quenching ROS (as it has been previously proposed by Benaroudj et al., 2001). In support of this mechanism for the protection of proteins from ROS-elicited oxidative damage by trehalose, we found that in pre-quiescent cells the *tsl1*Δ and *nth1*Δ mutations reduce oxidative carbonylation of cellular proteins (Figure 2A) but do not alter ROS levels (Figures 2B and 5B,C, process 5).

The oxidatively carbonylated protein species present in pre-quiescent WT cells are known to have a tendency to form insoluble aggregates that escape degradation and can compromise the cellular proteostasis network by inhibiting the proteasomal protein degradation machinery (Nyström, 2005; Hipkiss, 2006; Taylor and Dillin, 2011). By protecting cellular proteins from oxidative carbonylation (Figure 5A, process 5; see above), trehalose reduces the formation of insoluble protein aggregates prior to entry into senescence (Figure 5A, process 6). This indirect inhibitory effect of trehalose on protein aggregation supplements its direct inhibition by trehalose (Figure 5A, process 3; see above), which could shield the patches of exposed hydrophobic side chains of amino acids tending to promote aggregation of aberrantly folded and unfolded protein species (Figure 5A, process 4; see above).

Following entry into a quiescent state, a network of molecular chaperones in WT cells promotes a refolding of misfolded, partially folded, and unfolded protein species, either soluble or extracted from protein aggregates accumulated in pre-quiescent cells (Figure 5A, processes 7 and 8). This chaperone-assisted refolding of aberrantly folded protein species is the essential anti-aging process (Kikis et al., 2010; Chen et al., 2011; Lindquist and Kelly, 2011; Taylor and Dillin, 2011). By shielding the contiguous exposed hydrophobic side chains of amino acids that are abundant in misfolded, partially folded and unfolded protein species, trehalose in quiescent WT cells competes with molecular chaperones for binding with these patches of hydrophobic amino acid residues (Figure 5A, processes 9 and 10) known to be mandatory for enabling the chaperone-assisted refolding of aberrantly folded protein species (Kikis et al., 2010; Chen et al., 2011; Lindquist and Kelly, 2011; Taylor and Dillin, 2011). By interfering with this essential anti-aging process in quiescent WT cells, trehalose operates as a pro-aging compound (Figure 5A). In support of our hypothesis that this mechanism underlies the essential role of trehalose homeostasis in defining longevity of chronologically aging yeast under CR conditions (Figures 5B,C, processes 9 and 10) we found that (1) the *tsl1*Δ mutation reduces trehalose concentration following entry into quiescence (Figure 1B), decreases the extent of protein aggregation in quiescent cells (Figure 3) and extends yeast chronological lifespan (Figure 1D); and (2) the *nth1*Δ mutation elevates trehalose concentration following entry into quiescence (Figure 1C), increases the extent of protein aggregation in quiescent cells (Figure 3) and shortens yeast chronological lifespan (Figure 1E).

The major challenge now is to get a greater insight into the proposed mechanism underlying the essential role of trehalose homeostasis in defining longevity of chronologically aging yeast under lifespan-extending CR conditions. To address this challenge, many important questions need to be answered. What are the identities of oxidatively damaged proteins whose accumulation in pre-quiescent WT cells proliferating under CR conditions is reduced by genetic manipulations that elevate trehalose concentration prior to entry into quiescence (Figure 2A)? Are these proteins known for their essential role in defining longevity? Will genetic manipulations eliminating any of these proteins or altering their levels affect the chronological lifespan of yeast? What kind of proteins form insoluble aggregates that accumulate, in a trehalose-dependent fashion, in WT cells prior to and/or following entry into a quiescent state (Figure 3)? Are they known to be modifiers of lifespan in yeast? How will genetic manipulations eliminating any of these proteins or altering their levels influence longevity of chronologically aging yeast? Do oxidatively damaged and/or aggregated protein species concentrate in certain protein quality control compartments, such as the juxtanuclear quality control compartment, the insoluble protein deposit compartment and/or aggresome (Ben-Gedalya et al., 2011; Chen et al., 2011; Ben-Gedalya and Cohen, 2012), or are they randomly distributed throughout a cell prior to and/or following entry into quiescence? Does trehalose reside, permanently or temporarily, in any of these protein quality control compartments or is this osmolyte dispersed within a cell before and/or after it enters a quiescent state? What molecular chaperones constitute the proteostasis machinery whose ability to refold aberrantly folded proteins is compromised by trehalose in quiescent cells? We shall have to answer these important questions if we want to understand the complexity of the proteostasis network that defines longevity by sensing the dynamics of age-related changes in trehalose concentration.

## Conflict of Interest Statement

The authors declare that the research was conducted in the absence of any commercial or financial relationships that could be construed as a potential conflict of interest.

## References

[B1] Anderson R. M. Weindruch R. (2010). Metabolic reprogramming, caloric restriction and aging. Trends Endocrinol. Metab. 21, 134–141 10.1016/j.tem.2009.11.005 20004110 PMC2831168

[B2] Benaroudj N. Lee D. H. Goldberg A. L. (2001). Trehalose accumulation during cellular stress protects cells and cellular proteins from damage by oxygen radicals. J. Biol. Chem. 276, 24261–24267 10.1074/jbc.M101487200 11301331

[B3] Ben-Gedalya T. Cohen E. (2012). Quality control compartments coming of age. Traffic 13, 635–642 10.1111/j.1600-0854.2012.01330.x 22280095

[B4] Ben-Gedalya T. Lyakhovetsky R. Yedidia Y. Bejerano-Sagie M. Kogan N. M. Karpuj M. V. Kaganovich D. Cohen E. (2011). Cyclosporin-A-induced prion protein aggresomes are dynamic quality-control cellular compartments. J. Cell Sci. 124, 1891–1902 10.1242/jcs.077693 21558416

[B5] Boukh-Viner T. Guo T. Alexandrian A. Cerracchio A. Gregg C. Haile S. Kyskan R. Milijevic S. Oren D. Solomon J. Wong V. Nicaud J.-M. Rachubinski R. A. English A. M. Titorenko V. I. (2005). Dynamic ergosterol- and ceramide-rich domains in the peroxisomal membrane serve as an organizing platform for peroxisome fusion. J. Cell Biol. 168, 761–773 10.1083/jcb.200409045 15738267 PMC2171827

[B6] Chen B. Retzlaff M. Roos T. Frydman J. (2011). Cellular strategies of protein quality control. Cold Spring Harb. Perspect. Biol. 3, a004374 10.1101/cshperspect.a004374 21746797 PMC3140689

[B7] Chow T. Zukin R. S. (1983). Solubilization and preliminary characterization of mu and kappa opiate receptor subtypes from rat brain. Mol. Pharmacol. 24, 203–212 6310362

[B8] Colman R. J. Anderson R. M. Johnson S. C. Kastman E. K. Kosmatka K. J. Beasley T. M. Allison D. B. Cruzen C. Simmons H. A. Kemnitz J. W. Weindruch R. (2009). Caloric restriction delays disease onset and mortality in rhesus monkeys. Science 325, 201–204 10.1126/science.1173635 19590001 PMC2812811

[B9] Elbein A. D. Pan Y. T. Pastuszak I. Carroll D. (2003). New insights on trehalose: a multifunctional molecule. Glycobiology 13, 17R–27R 10.1093/glycob/cwg047 12626396

[B10] Evans E. A. Gilmore R. Blobel G. (1986). Purification of microsomal signal peptidase as a complex. Proc. Natl. Acad. Sci. U.S.A. 83, 581–585 10.1073/pnas.83.17.6387 3511473 PMC322907

[B11] Fontana L. Partridge L. Longo V. D. (2010). Extending healthy life span – from yeast to humans. Science 328, 321–326 10.1126/science.1172539 20395504 PMC3607354

[B12] François J. Parrou J. L. (2001). Reserve carbohydrates metabolism in the yeast *Saccharomyces cerevisiae*. FEMS Microbiol. Rev. 25, 125–145 10.1111/j.1574-6976.2001.tb00574.x 11152943

[B13] Gems D. Doonan R. (2009). Antioxidant defense and aging in *C. elegans*: is the oxidative damage theory of aging wrong? Cell Cycle 8, 1681–1687 10.4161/cc.8.11.8595 19411855

[B14] Gidalevitz T. Prahlad V. Morimoto R. I. (2011). The stress of protein misfolding: from single cells to multicellular organisms. Cold Spring Harb. Perspect. Biol. 3, a009704 10.1101/cshperspect.a009704 21536706 PMC3098679

[B15] Goldberg A. A. Bourque S. D. Kyryakov P. Gregg C. Boukh-Viner T. Beach A. Burstein M. T. Machkalyan G. Richard V. Rampersad S. Cyr D. Milijevic S. Titorenko V. I. (2009). Effect of calorie restriction on the metabolic history of chronologically aging yeast. Exp. Gerontol. 44, 555–571 10.1016/j.exger.2009.06.001 19539741

[B16] Goldberg A. A. Richard V. R. Kyryakov P. Bourque S. D. Beach A. Burstein M. T. Glebov A. Koupaki O. Boukh-Viner T. Gregg C. Juneau M. English A. M. Thomas D. Y. Titorenko V. I. (2010). Chemical genetic screen identifies lithocholic acid as an anti-aging compound that extends yeast chronological life span in a TOR-independent manner, by modulating housekeeping longevity assurance processes. Aging 2, 393–414 20622262 10.18632/aging.100168 PMC2933888

[B17] Greer E. L. Brunet A. (2008). Signaling networks in aging. J. Cell Sci. 121, 407–412 10.1242/jcs.021519 18256383

[B18] Greer E. L. Brunet A. (2009). Different dietary restriction regimens extend lifespan by both independent and overlapping genetic pathways in *C. elegans*. Aging Cell 8, 113–127 19239417 10.1111/j.1474-9726.2009.00459.x PMC2680339

[B19] Guarente L. P. Partridge L. Wallace D. C. (2008). Molecular Biology of Aging. Cold Spring Harbor: Cold Spring Harbor Laboratory Press

[B20] Harman D. (1956). Aging: a theory based on free radical and radiation chemistry. J. Gerontol. 11, 298–300 13332224 10.1093/geronj/11.3.298

[B21] Harman D. (1972). The biologic clock: the mitochondria? J. Am. Geriatr. Soc. 20, 145–147 5016631 10.1111/j.1532-5415.1972.tb00787.x

[B22] Hekimi S. Lapointe J. Wen Y. (2011). Taking a “good” look at free radicals in the aging process. Trends Cell Biol. 21, 569–576 10.1016/j.tcb.2011.06.008 21824781 PMC4074523

[B23] Hipkiss A. R. (2006). Accumulation of altered proteins and ageing: causes and effects. Exp. Gerontol. 41, 464–473 10.1016/j.exger.2006.03.004 16621390

[B24] Jain N. K. Roy I. (2009). Effect of trehalose on protein structure. Protein Sci. 18, 24–36 19177348 10.1002/pro.3 PMC2708026

[B25] Jain N. K. Roy I. (2010). Trehalose and protein stability. Curr. Protoc. Protein Sci. 59, 49.1–4.9.12. 10.1002/0471140864.ps0409s59 20155732

[B26] Kaeberlein M. (2010). Lessons on longevity from budding yeast. Nature 464, 513–519 10.1038/nature09046 20336133 PMC3696189

[B27] Kenyon C. (2001). A conserved regulatory system for aging. Cell 105, 165–168 10.1016/S0092-8674(01)00306-3 11336665

[B28] Kenyon C. (2005). The plasticity of aging: insights from long-lived mutants. Cell 120, 449–460 10.1016/j.cell.2005.02.002 15734678

[B29] Kenyon C. (2011). The first long-lived mutants: discovery of the insulin/IGF-1 pathway for ageing. Philos. Trans. R. Soc. Lond. B Biol. Sci. 366, 9–16 10.1098/rstb.2010.0276 21115525 PMC3001308

[B30] Kenyon C. J. (2010). The genetics of ageing. Nature 464, 504–512 10.1038/nature08980 20336132

[B31] Kikis E. A. Gidalevitz T. Morimoto R. I. (2010). Protein homeostasis in models of aging and age-related conformational disease. Adv. Exp. Med. Biol. 694, 138–159 10.1007/978-1-4419-7002-2_11 20886762 PMC3402352

[B32] Kirkwood T. B. L. (2008). Understanding ageing from an evolutionary perspective. J. Intern. Med. 263, 117–127 10.1111/j.1365-2796.2007.01901.x 18226090

[B33] Lapointe J. Hekimi S. (2010). When a theory of aging ages badly. Cell. Mol. Life Sci. 67, 1–8 10.1007/s00018-009-0138-8 19730800 PMC4053417

[B34] Lin S. S. Manchester J. K. Gordon J. I. (2001). Enhanced gluconeogenesis and increased energy storage as hallmarks of aging in *Saccharomyces cerevisiae*. J. Biol. Chem. 276, 36000–36007 10.1074/jbc.M104989200 11461906

[B35] Lindquist S. L. Kelly J. W. (2011). Chemical and biological approaches for adapting proteostasis to ameliorate protein misfolding and aggregation diseases: progress and prognosis. Cold Spring Harb. Perspect. Biol. 3, a004507 10.1101/cshperspect.a004507 21900404 PMC3225948

[B36] Madeo F. Fröhlich E. Fröhlich K.-U. (1997). A yeast mutant showing diagnostic markers of early and late apoptosis. J. Cell Biol. 139, 729–734 10.1083/jcb.139.3.729 9348289 PMC2141703

[B37] Mair W. Dillin A. (2008). Aging and survival: the genetics of life span extension by dietary restriction. Annu. Rev. Biochem. 77, 727–754 10.1146/annurev.biochem.77.061206.171059 18373439

[B38] Masoro E. J. (2002). Caloric Restriction: A Key to Understanding and Modulating Aging. Amsterdam: Elsevier

[B39] Masoro E. J. Austad S. N. (2011). Handbook of the Biology of Aging, 7th Edn. Amsterdam: Academic Press

[B40] Mir S. S. Fiedler D. Cashikar A. G. (2009). Ssd1 is required for thermotolerance and Hsp104-mediated protein disaggregation in *Saccharomyces cerevisiae*. Mol. Cell. Biol. 29, 187–200 10.1128/MCB.02271-07 18936161 PMC2612483

[B41] Morimoto R. I. Selkoe D. J. Kelly J. W. (2012). Protein Homeostasis. Cold Spring Harbor: Cold Spring Harbor Laboratory Press

[B42] Narasimhan S. D. Yen K. Tissenbaum H. A. (2009). Converging pathways in lifespan regulation. Curr. Biol. 19, R657–R666 10.1016/j.cub.2009.06.013 19674551 PMC3109866

[B43] Nyström T. (2005). Role of oxidative carbonylation in protein quality control and senescence. EMBO J. 24, 1311–1317 10.1038/sj.emboj.7600599 15775985 PMC1142534

[B44] Parsell D. A. Kowal A. S. Singer M. A. Lindquist S. (1994). Protein disaggregation mediated by heat-shock protein Hsp104. Nature 372, 475–478 10.1038/372475a0 7984243

[B45] Pérez V. I. Bokov A. Van Remmen H. Mele J. Ran Q. Ikeno Y. Richardson A. (2009). Is the oxidative stress theory of aging dead? Biochim. Biophys. Acta 1790, 1005–1014 10.1016/j.bbagen.2009.06.003 19524016 PMC2789432

[B46] Pluskal T. Hayashi T. Saitoh S. Fujisawa A. Yanagida M. (2011). Specific biomarkers for stochastic division patterns and starvation-induced quiescence under limited glucose levels in fission yeast. FEBS J. 278, 1299–1315 10.1111/j.1742-4658.2011.08050.x 21306563 PMC3123465

[B47] Ristow M. Zarse K. (2010). How increased oxidative stress promotes longevity and metabolic health: the concept of mitochondrial hormesis (mitohormesis). Exp. Gerontol. 45, 410–418 10.1016/j.exger.2010.03.014 20350594

[B48] Sanz A. Fernández-Ayala D. J. Stefanatos R. K. Jacobs H. T. (2010). Mitochondrial ROS production correlates with, but does not directly regulate lifespan in Drosophila. Aging 2, 200–223 20453260 10.18632/aging.100137 PMC2880708

[B49] Singer M. A. Lindquist S. (1998a). Multiple effects of trehalose on protein folding in vitro and in vivo. Mol. Cell 1, 639–648 10.1016/S1097-2765(00)80064-7 9660948

[B50] Singer M. A. Lindquist S. (1998b). Thermotolerance in *Saccharomyces cerevisiae*: the Yin and Yang of trehalose. Trends Biotechnol. 16, 460–468 10.1016/S0167-7799(98)01251-7 9830154

[B51] Tao H. Liu W. Simmons B. N. Harris H. K. Cox T. C. Massiah M. A. (2010). Purifying natively folded proteins from inclusion bodies using sarkosyl, triton X-100, and CHAPS. BioTechniques 48, 61–64 10.2144/000113304 20078429

[B52] Tavernarakis N. (2010). Protein Metabolism and Homeostasis in Aging. Austin: Landes Bioscience 20886751

[B53] Taylor R. C. Dillin A. (2011). Aging as an event of proteostasis collapse. Cold Spring Harb. Perspect. Biol. 3, a004440 10.1101/cshperspect.a004440 21441594 PMC3101847

[B54] Titorenko V. I. Smith J. J. Szilard R. K. Rachubinski R. A. (1998). Pex20p of the yeast Yarrowia lipolytica is required for the oligomerization of thiolase in the cytosol and for its targeting to the peroxisome. J. Cell Biol. 142, 403–420 10.1083/jcb.142.2.403 9679140 PMC2133052

[B55] Trevisol E. T. Panek A. D. Mannarino S. C. Eleutherio E. C. (2011). The effect of trehalose on the fermentation performance of aged cells of *Saccharomyces cerevisiae*. Appl. Microbiol. Biotechnol. 90, 697–704 10.1007/s00253-010-3053-x 21243352

[B56] Wang J. Jiang J. C. Jazwinski S. M. (2010). Gene regulatory changes in yeast during life extension by nutrient limitation. Exp. Gerontol. 45, 621–631 10.1016/j.exger.2010.07.004 20178842 PMC2879456

[B57] Weindruch R. Walford R. L. (1988). The Retardation of Aging and Disease by Dietary Restriction. Springfield: Thomas

